# Neuroprotective Effect of Dexmedetomidine against Postoperative Cognitive Decline via NLRP3 Inflammasome Signaling Pathway

**DOI:** 10.3390/ijms23158806

**Published:** 2022-08-08

**Authors:** Inja Cho, Bon-Nyeo Koo, So Yeon Kim, Sujung Park, Eun Jung Kim, Eun Hee Kam, Jeongmin Kim

**Affiliations:** 1 Department of Anesthesiology and Pain Medicine, Yonsei University College of Medicine, 50-1, Yonsei-ro, Seodaemun-gu, Seoul 03722, Korea; 2 Anesthesia and Pain Research Institute, Yonsei University College of Medicine, 50-1, Yonsei-ro, Seodaemun-gu, Seoul 03722, Korea

**Keywords:** antioxidant, dexmedetomidine, inflammation, NLRP3 inflammasome, reactive oxygen species, postoperative cognitive dysfunction

## Abstract

Dexmedetomidine (Dex), widely used as a sedative in surgical procedures and intensive care units, induces sympatholytic, anxiolytic, analgesic, and sedative effects. Postoperative cognitive dysfunction (POCD) is routinely observed in postoperative care following surgery and general anesthesia. The NLRP3 inflammasome complex plays a critical role in innate immune response by detecting pathogenic microorganisms and activating pro-inflammatory cytokines. Although there are numerous protective effects of Dex among the neurological diseases, specific mechanisms including NLRP3 inflammasome-mediated neuroinflammation via oxidative stress response in a POCD model are not fully understood. Here, we investigated whether Dex exhibits neurocognitive effects through the NLRP3 inflammasome signaling in a POCD mouse model using a neurobehavioral test and ELISA analysis. We also confirmed the level of oxidative stress-related response in the in vitro system in the POCD model. Furthermore, we evaluated the NLRP3 inflammasome complex by immunoprecipitation analysis. In summary, the results of the present study indicated that Dex showed a neuroprotective effect in the POCD model by reducing oxidative stress response through NLRP3 inflammasome-mediated neuroinflammation.

## 1. Background

Dexmedetomidine (Dex), an α2-adrenoceptor agonist, is widely used as a sedative agent in surgical procedures and intensive care units as it induces sympatholytic, analgesic, and sedative effects [1,2]. Few studies have demonstrated the protective effects of Dex against ischemia-reperfusion injury, cardiovascular impairment, respiratory, and acute lung injury [3,4]. Similarly, it was reported that Dex regulates the signaling molecules, such as ERK and PI3K/Akt, in vascular endothelial cells and glioma cells [5,6]. In addition, Dex exerts an anti-inflammatory effect by reducing the expression of inflammatory cytokines, such as IL-6 and TNF-α [7,8]. Many biological functions of Dex have been demonstrated in diverse pathological conditions and signaling pathways.

Postoperative cognitive dysfunction (POCD) is commonly presented in postoperative care following surgery and general anesthesia. Transient cognitive impairment manifested due to POCD can contribute to severe cognitive defects in the long term [9] and lead to dementia [10]. Although age, surgical duration, infection, and inflammation have been identified as the factors that lead to POCD, the exact pathophysiological mechanism of POCD pathogenesis and optimal strategies for treatment or prevention of POCD remain unclear. Recently, neuroinflammation caused by systemic inflammation following surgery has been suggested as a key factor for the development of POCD.

The nucleotide-binding oligomerization domains (NODs) are components of NOD-like receptors (NLRs), which play a vital role in innate immunity [11,12]. The NLR family pyrin domain containing 3 (NLRP3) inflammasome consists of NLRP3 protein, apoptosis-associated speck-like protein (ASC), and pro-caspase-1, which regulates IL-1β activation and secretion by active caspase-1. Thus, the dysregulation of the NLRP3 inflammasome is associated with various diseases, including ischemic stroke, diabetes, multiple sclerosis, atherosclerosis, Alzheimer’s disease, and other inflammatory-related diseases [13,14,15,16]. In addition, recent studies have revealed that dysregulation of NLRP3 inflammasome was associated with cognitive impairment [17], and the impairment could be reversed by inhibiting the NLRP3 inflammasome [18]. Previous studies have demonstrated that Dex regulates NLRP3 inflammasome against diverse pathologic conditions, such as osteoarthritis, lung injury and ischemic stroke [19,20]. The aim of the present study is to investigate the mechanism by which Dex alleviates neurocognitive impairment in the POCD model though NLRP3 inflammasome mediated neuroinflammation via oxidative stress.

## 2. Results

### 2.1. Identification of SMA Surgery-Induced POCD Mice Model 

**Figure 1:**
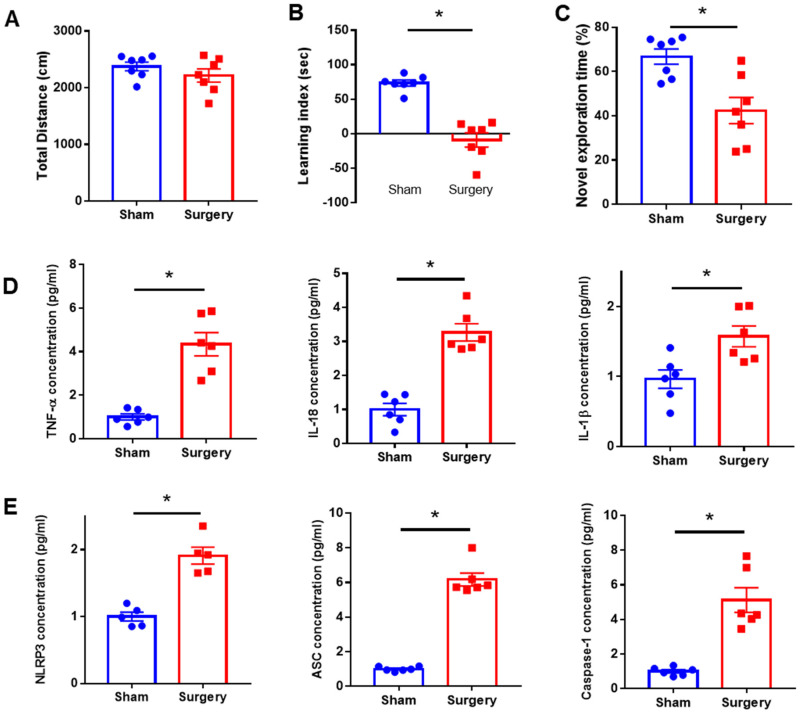
Identification of the POCD model. Assessment of neurobehavioral abilities of SMA surgery mice (n = 6 per group). (**A**) Total distance travelled in the open field test. (**B**) Learning index (transfer latency time to enter the closed arm during the training period—transfer latency time to enter the closed arm during the test period) in the elevated plus maze test. (**C**) Discrimination time rate for novel objects in the test phase of the novel objective recognition test. The level of inflammatory cytokines and NLRP3 inflammasome component in SMA surgery mice. (**D**) IL-1β, IL-18, and TNF-α and (**E**) NLRP3, ASC, and caspasei-1 in the hippocampus. Sham, Sham control group; SMA, Superior mesenchymal artery surgery group; * *p* < 0.05 compared to the sham.

To investigate the neurobehavior impairments caused by surgery, we performed a neurobehavior test post day 5. In the OFT, the total distance traveled did not show significant difference among groups (Figure 1A). In the EPM test, the learning index was significantly reduced in the surgery group compared to the sham control group (Figure 1B). In addition, the discrimination time rate for novel objects in NORT was also significantly decreased in the surgery group compared to the sham control (Figure 1C).

To evaluate the alteration of inflammatory response and NLRP3 inflammasome in the hippocampus, we measured the level of inflammatory cytokines, such as TNF-α, IL-18, IL-1β, and the NLRP3 inflammasome components including NLRP3, ASC, and caspase-1. IL-1β, IL-18, and TNF-α levels were significantly increased in the hippocampus in the surgery group (Figure 1D). The levels of NLRP3, ASC, and caspase-1 were also significantly increased in the surgery group compared to the sham control (Figure 1E). Consequently, surgery-induced POCD mice showed cognitive impairment and an elevated inflammatory response with increased NLRP3 inflammasome activation. 

### 2.2. Effect of Dex on Neurobehavior in POCD Mice Model

**Figure 2:**
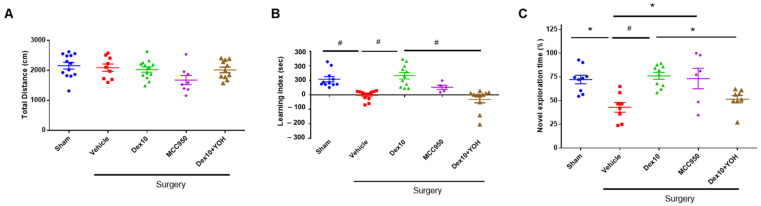
The effect of Dex in POCD model. Assessment of the neurobehavioral abilities of SMA surgery mice. Number of mice for each group: Sham n = 9~13; Vehicle n = 9~13; Dex 10 n = 10~13; MCC950 n = 5~8; Dex + YOH n = 8~11. (**A**) Total distance travelled in the open field test. (**B**) Learning index (transfer latency time to enter the closed arm during the training period—transfer latency time to enter the closed arm during the test period) in the elevated plus maze test. (**C**) Discrimination time rate for novel objects in the test phase of the novel objective recognition test Sham, Sham control group; SMA, Superior mesenchymal artery surgery group; *, *p* < 0.05; #, *p* < 0.001.

To analyze the effect of Dex in the POCD mice model, we injected mice with Dex, MCC 950, and YOH for 5 days. In an open field test, the total distance traveled did not differ significantly among the groups (Figure 2A). In the surgery group treated with Dex, the learning index was significantly increased compared to the surgery group treated with saline (Veh) in the EPM test (Figure 2B). In NORT, the discrimination time rate for novel objects was also significantly increased in the Dex group compared to the Veh group (Figure 2C).

The rate of exploring time for a new object was also increased in the surgery group treated with MCC950 (as an NLRP3 inhibitor) compared to the Veh group. To confirm that the behavioral change was due to Dex treatment, YOH (as a Dex antagonist) was administered. Interestingly, the learning index and the rate of exploring time for a new object in the surgery group treated with Dex + YOH was significantly decreased compared to the Dex only group. These results suggest that treatment with Dex improves cognitive impairment in POCD mice, which might involve the NLRP3 inflammasome pathway and the alpha-2 adrenergic receptor.

### 2.3. Effect of Dex on Inflammatory Response in the Hippocampus

**Figure 3:**
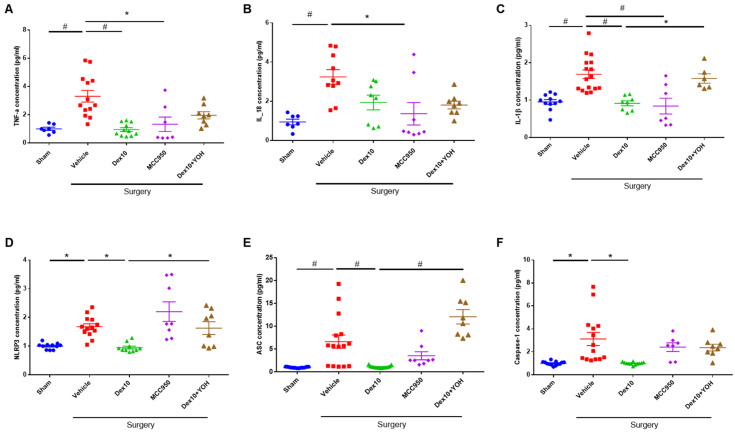
The anti-inflammatory effects of Dex against the POCD model. Number of mice for each group: Sham n = 7~11; Vehicle n = 10~15; Dex 10 n = 8~11; MCC950 n =  7~8; Dex + YOH n = 6~8. Assessment of the level of inflammatory cytokines and NLRP3 inflammasome components by ELISA analysis. The level of (**A**) TNF-α, (**B**) IL-18, (**C**) IL-1β and (**D**) NLRP3, (**E**) ASC, and (**F**) caspase-1 in the hippocampus, *, *p* < 0.05; #, *p* < 0.001.

ELISA analysis results showed that the levels of TNF-α, IL-18, and IL-1β (vehicle) were increased after surgery. However, the levels significantly decreased in the Dex and MCC650-treated groups. The level of NLRP3 was significantly decreased in the Dex group but not in the MCC950 group compared to the Veh group. Interestingly, the Dex + YOH group showed significantly increased levels of IL-1β, NLRP3, and ASC compared to the Dex group post-surgery (Figure 3). These results suggested that Dex has an anti-inflammatory effect by regulating the NLRP3 inflammasome.

### 2.4. Expression of NLRP3 Inflammasome in the BV2 Cells

**Figure 4:**
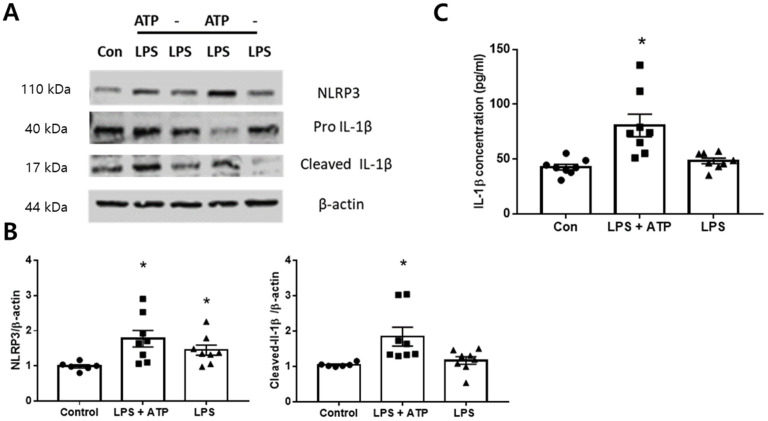
NLRP3 inflammasome activation in BV2 cells. Activation of NLRP3 inflammasome model induced by treatment with LPS and ATP (n = 6~8 per group). (**A**) Western blot analysis of NLRP3 and IL-1β in BV2 cell lysate, (**B**) Quantitative data showing protein expressions, (**C**) The level of IL-1β in the cell culture medium, * *p* < 0.05 compared to the control.

The activation of the NLRP3 inflammasome involves two phases. The first phase is the priming phase, which results in the upregulation of inactive NLRP3 protein. The second phase is the activation phase, which involves the assembly of NLRP3 and ASC into the inflammasome complex initiated by excess ATP, and subsequent maturation and release of IL-1β. To activate the NLRP3 inflammasome, BV2 cells were incubated with lipopolysaccharide (LPS, 1 mg/mL) for 3 h and 5 μM concentrations of ATP for 30 min. Increased expression of NLRP3 was indeed detected in the LPS + ATP group compared to the control, and an increased level of mature IL-1β was detected in the cell lysate (Figure 4A). Increased levels of IL-1β in the culture medium were also detected in the LPS + ATP group (Figure 4B). These results indicate that the successful in vitro POCD model was established. 

### 2.5. Dex Inhibited NLRP3 Inflammasome Activation In Vitro

**Figure 5:**
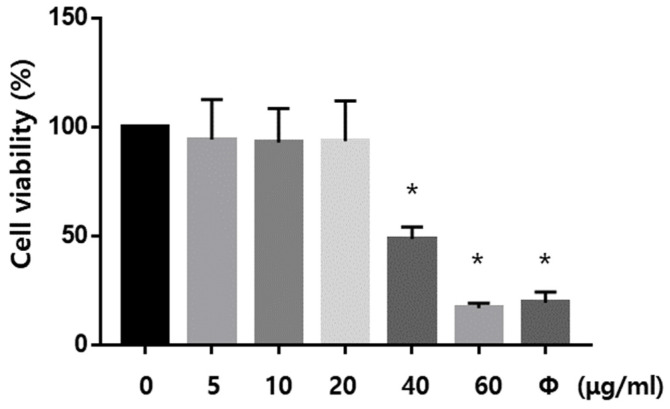
Determination of Dexmedetomidine concentration in BV2 cells (n = 5 per group). Cell viability was assessed to obtain optimum concentration of Dex. Ø, treated DMSO for negative control. * *p* < 0.05 compared to the 0 μg/mL of Dexmedetomidine.

To determine an optimum non-cytotoxic dose of Dex, BV2 cells were treated with various concentrations of Dex (0, 5, 10, 20, 40, 60 μg/mL) for 24 h before exposing them to LPS and ATP, and the cell viability was analyzed using the MTT assay. The subsequent experiments were performed 20 μg/mL of Dex because at 40 μg/mL or more of Dex showed severe cell toxicity (Figure 5).

**Figure 6:**
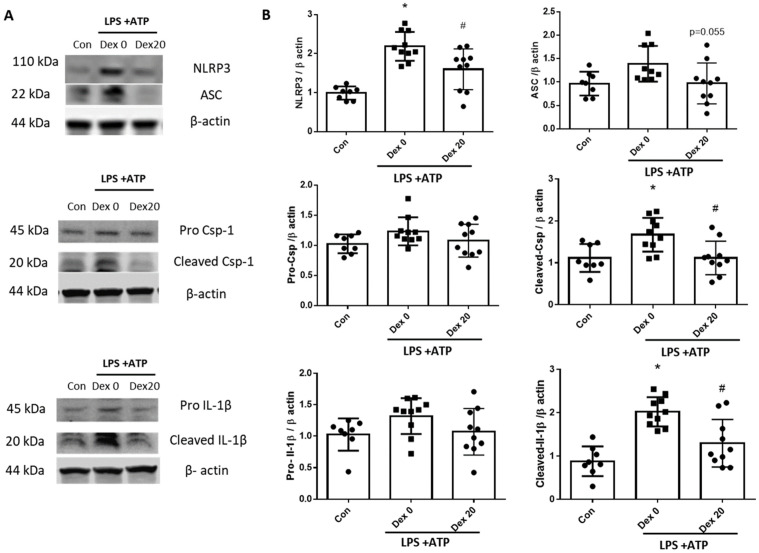
Downregulation of NLRP3 inflammasome complex by Dex treatment (n = 8~10 per group). (**A**) Western blot analysis of NLRP3, ASC, Caspase-1, and IL-1β in BV2 cell lysate, (**B**) quantitative data showing protein expressions, *, *p* < 0.05 compared to the con; #, *p* < 0.05 compared to the Dex 0.

To determine the protective effect of Dex, cells were pretreated with Dex for 24 h before exposing them to LPS and ATP. The effect of Dex was confirmed using western blotting (Figure 6A). The normal BV2 cells were used as a control. In cells induced by LPS + ATP without Dex-pretreatment, the expression of the NLRP3 inflammasome components including ASC, caspase-1, and IL-1β was significantly higher than the control group. Matured IL-1β form was also significantly increased in the LPS + ATP group compared to the control group. In contrast, the expression of NLRP3, ASC, caspase-1, and cleaved-IL-1β were significantly decreased in the Dex-pretreated group compared to the LPS + ATP group without Dex-pretreatment (Figure 6B).

### 2.6. Dex Ameliorated the Oxidative Stress-Induced Neuronal Cell Death In Vitro

**Figure 7:**
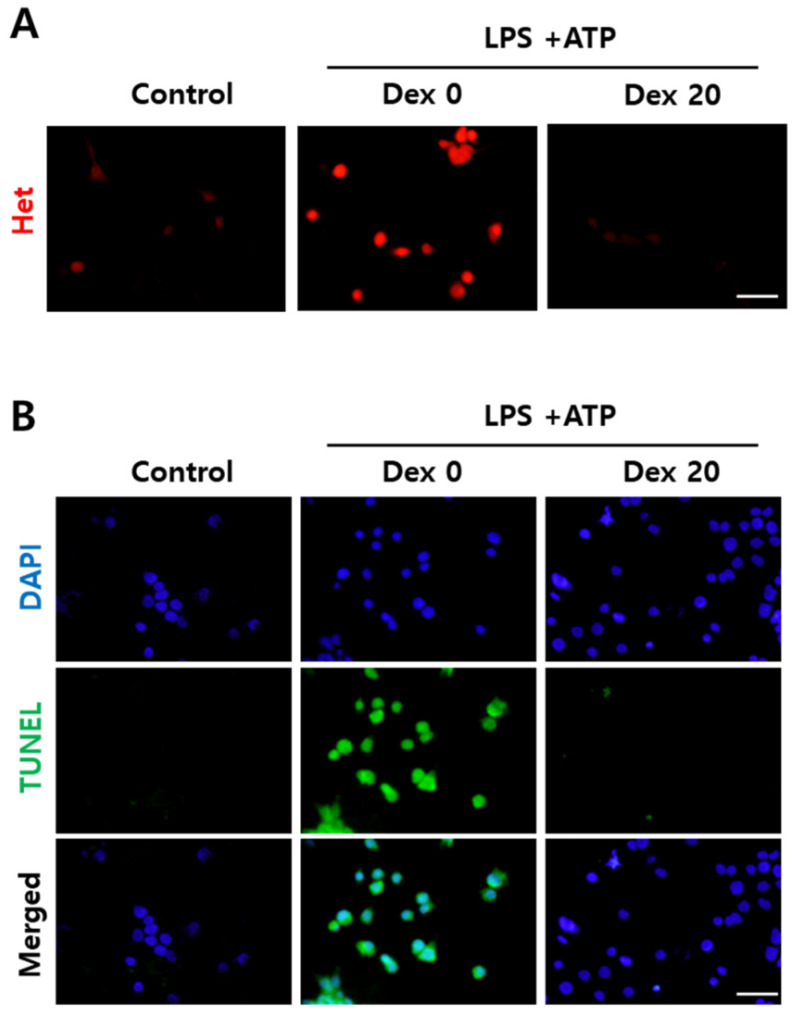
Protective effect of Dex on oxidative stress-induced cell death in PC12 cells. PC12 cells were stimulated with conditioned medium from BV2 cells treated with LPS and ATP or Dex. Representative image of (**A**) intracellular ROS detection after a 16 h incubation period with conditioned medium and (**B**) cell death detection after 24 h incubation with conditioned medium. Scale bar = 50 μm.

To analyze the effect of oxidative stress and cell death, PC12 cells were exposed to the CM from BV2 for 16 and 24 h, respectively. In LPS + ATP induced cells without Dex pretreatment (Dex0), oxidative stress (red signal; oxidized Het) was markedly increased compared to the control group (Figure 7A). The number of TUNEL-positive cells were also significantly increased in the Dex0 group compared to the control (Figure 7B). However, excessive ROS and the number of TUNEL-positive cells decreased markedly in the Dex treated group (Figure 7). These results suggest that Dex protects the cells against oxidative stress-related damage.

### 2.7. Dex Inhibits NLRP3 Inflammasome Activation In Vitro

**Figure 8:**
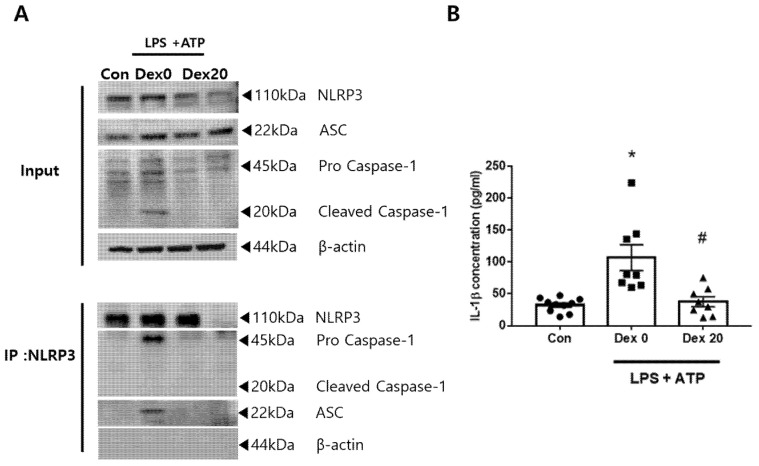
Dexmedetomidine attenuates activation of NLRP3 inflammasome in BV2 cells. BV2 cells were untreated or stimulated with LPS or LPS + ATP or Dex (n = 7~8 per group). (**A**) Immunoprecipitation analysis of the NLRP3 inflammasome interacting components. NLRP3, ASC, and Caspase-1. (**B**) IL-1β release in conditioned medium was measured, *, *p* < 0.05 compared to the con; #, *p* < 0.05 compared to the Dex 0.

To investigate whether DEX can directly regulate the NLRP3 inflammasome, we assessed the formation of the NLRP3 inflammasome, which consists of NLRP3, ASC, and pro-caspase 1, using IP analysis in Bv2 cells. The results revealed that the NLRP3 pull-down in LPS + ATP-induced cells without Dex group (Dex 0) showed a higher amount of ASC and pro-caspase-1 compared to the control, indicating that the NLRP3 inflammasome formation was increased after LPS + ATP stimulation, which was inhibited by Dex treatment (Figure 8A). Moreover, in the input, cleaved caspase-1 was only detected in the Dex0 group. In addition, IL-1β in the cell culture supernatant was significantly increased in the Dex0 group compared to the control, which was reversed by Dex-pretreatment. These results indicated that Dex exerts an anti-inflammatory effect by directly suppressing the activation of NLRP3 inflammasome induced by LPS + ATP in BV2 cells.

## 3. Discussion

We confirmed that the surgery induced cognitive impairment and increased the levels of pro-inflammatory cytokines by activating the NLRP3 inflammasome. DEX administration improved the cognitive impairment and reduced pro-inflammatory cytokines in the POCD model by regulating NLRP3 and ASC expression. MCC950 also improved cognitive impairment in the surgery model but did not regulate NLRP3 expression. YOH, a selective α2 antagonist, reversed the neuroprotective effect of Dex in the surgery model. In addition, we confirmed that Dex administration exerts neuroprotective effects and improves cognitive impairment by directly regulating NLRP3 inflammasome activation in an in vitro POCD model.

POCD is manifested by cognitive dysfunction following the surgery, which leads to increased morbidity and mortality. Inflammation and triggered immune response are the hallmarks of POCD [21]. Analgesic agents reduce the response to surgical stress and improve the tolerance of patients. Dex has been widely used in clinical trials [22]. Dex has a high affinity and selectivity for the α2-adrenoceptors and induces sympatholytic effect, such as a lower mean arterial blood pressure and heart rate, through the activation of α2-adrenoceptors in the central nervous system [3,23]. Furthermore, many clinical studies reported multiple benefits of Dex, including reduction in the severity of surgical stress and improvement in the tolerance of patients after various surgeries [24,25,26,27,28,29,30]. Inflammatory cytokines are implicated in the outcome of diverse pathological conditions. Recent studies showed that Dex could enhance HMGB1 resolution in mice, suggesting its ability to reduce inflammation [31]. In addition, many studies have reported that Dex could ameliorate the release of inflammatory cytokines [20,32] and inhibit the NLRP3 inflammasome activation. In the present study, we also confirmed that Dex could inhibit the increase in the levels of pro-inflammatory cytokines and the activation of NLRP3 inflammasome in POCD conditions. With the administration of MCC950, similar results were obtained after surgery. MCC950 was introduced as a highly potent NLRP3 inhibitor, which blocks the formation of NLRP3 inflammasome [18]. It was reported that the administration of MCC950 could inhibit the level of NLRP3, ASC, and IL-1β in hippocampus and ameliorate cognitive impairment in db/db mice [33]. In aged mice exposed to isoflurane, MCC950 treatment also ameliorated cognitive impairment [34]. This study also revealed that MCC950 treatment decreased the level of IL-1β and ameliorated cognitive impairment in the surgery model. However, the level of NLRP3 expression was not affected. These results are consistent with a previous report suggesting that MCC950 blocks the assembly and formation of NLRP3, ASC, and caspase-1complex [15].

Additionally, YOH, an α2-AR inhibitor, was used in our study to inhibit the effects of Dex. A previous study showed that YOH prevents the attenuation of inflammatory cytokines mediated by Dex [35]. Similarly, we observed that YOH administration reversed the effect of Dex of the inflammatory response and cognitive impairment. Moreover, the administration of YOH with DEX increased the level of NLRP3 and ASC expression compared to the Dex only group, consistent with results obtained in previous studies [36,37]. Therefore, present study confirmed that the effects of Dex on the POCD condition are executed by directly regulating level of NLRP3 and ASC expression and activation. In addition, we observed that Dex revealed a neuroprotective effect and an anti-inflammatory effect in the POCD model. Consistent with our results, a previous study reported that Dex prevented cell death induced by ischemia/ reperfusion injury by regulating the matrix metalloproteinase, Bax expression, and caspase activation in neuronal cells [38]. Dex was also shown to exert a neuroprotective effect in the ischemia or tissue hypoxia model by activating the mitochondrial ATP-sensitive potassium channel [39]. Dex also protected PC12 cells against lidocaine-induced apoptosis by increasing the levels of Bcl2 and inhibiting caspase 3 activation [40]. In addition, Dex prevented apoptosis in lung alveolar epithelial cells induced by H2O2 [41] and glutamate-induced cytotoxicity by reducing the malondialdehyde and augmenting superoxide dismutase activity [42]. Based on our in vitro results, the increased oxidative stress and associated neuronal cell deaths induced through the NLRP3 inflammasome activation by BV2 cells were eventually ameliorated by the Dex administration. These results confirmed that Dex exhibited the neuroprotective effect of the POCD model by reducing the oxidative stress response through NLRP3 inflammasome-mediated neuroinflammation.

However, there are several limitations to this study. The major aim of this study was to investigate the effect of Dex through NLRP3 signaling on a POCD model. ROS generation partly depends on the activation of NLRP3 inflammasomes [43,44,45]. We could not confirm the effect of DEX on ROS regulation in vivo. Alternatively, we observed that the effect of DEX on oxidative stress-induced neuronal cell death was prevented by regulating the NLRP3 inflammasome complex in PC12 cells. Oxidative stress has also been proposed as the possible cause for neurodegenerative and neurocognitive diseases leading to POCD and delirium [46]. It has been reported that mitochondrial oxidative stress is one of the crucial factors involved in NLRP3 inflammasome activation [44]. It is worth investigating whether the protective effect of Dex through the NLRP3 inflammasome pathway is also related to the regulation of oxidative stress. Therefore, further studies are required to examine diverse oxidative parameters and its scavenger system, such as SOD and catalase, for a complete understanding of the neuroprotective effect exerted by Dex.

## 4. Methods and Materials

### 4.1. Animals

All procedures involving animals were approved by the Institutional Animal Care and Use Committee of Yonsei University Health System (IACUC Approval number, 2018-0068), certified by the Association for Assessment and Accreditation of Laboratory Animal Care International (AAALAC). Adult (9–11 weeks) male C57BL/6 (Orient, Seongnam, Gyeonggi-do, Korea) mice were used for in vivo experiments. The mice were housed in groups of five per cage on a 12-h light/dark cycle with food and water available ad libitum.

### 4.2. Study Groups and Surgical Experimental Protocol

In experiment 1, the mice were randomly divided into two sets to investigate alteration of neurobehavioral abilities, inflammatory response and NLRP3 inflammasome after surgery: Sham vs. superior mesenteric artery (SMA) surgery (n = 6). In experiment 2, the mice were randomly divided into five groups as follows: Sham, Vehicle, Dex, MCC950, and Dex + YOH (n = 6~13 per group). The Sham group was anaesthetized with isoflurane without any surgical procedure. For the POCD model, the major abdominal surgery was mimicked. The surgical procedure was performed as described previously with some modifications [47]. Briefly, mice were anesthetized with 4% isoflurane and were placed on a heating pad during anesthesia to prevent hypothermia. After incision of the peritoneum, the superior mesenteric artery was clipped to mimic the abdominal surgery. Concomitantly, small intestine was exposed and jejunum, the part of small intestine, was rubbed for 30 s, and the procedure was repeated thrice with 1 min (min) interval to mimic the major abdominal surgery. The small intestine and exteriorized abdominal muscles were placed back into the peritoneal cavity and closed using sutures [48,49]. Dex was administered, at a dose of 10 µg/kg, i.p., 5 min before inducing anesthesia. Yohimbine hydrochloride (YOH, as an α2-adrenoceptor inhibitor, 2.5 µg/kg, i.p.) and MCC950 (as NLRP3 blocker, 10 mg/kg, i.p.) were administered 1 h before Dex treatment. All reagents were dissolved in the saline and vehicle group were injected with the same amount (100 μL) of the saline. 

### 4.3. Neurobehavioral Test

Cognitive ability was assessed using three different types of neurobehavioral tests five days after surgery. An open field test (OFT) was performed to assess general activity and locomotor functions. Mice were placed in a square open-field arena (40 cm × 40 cm × 40 cm), and the behavior was recorded for 5 min by recording the total distance moved. 

For assessing the learning memory function, an elevated plus maze (EPM) that consisted of two open arms (31 cm × 6 cm × 1 cm) and two enclosed arms (31 cm × 6 cm × 15 cm) with a central open square area (5 cm × 5 cm × 1 cm) and 50 cm height was performed and recorded time to enter the closed arms during allowed to explore for 5 min. Based on the natural aversion of mice to high and open spaces, the learning index is calculated as the difference in the entering time (in which the mouse first moves from the open arms to the closed arm) of the training period and entering time (in which the mouse first moves from the open arms to closed arm) of test period  =  learning index [50].

To evaluate the hippocampal-dependent recognition memory function, a novel object recognition test (NORT) was adopted in a square arena (40 cm × cm 40 × 40 cm) for 5 min [51]. In the familiarization phase, the mice were allowed to explore two same objects (● + ●) for 5 min. In the test phase, one object was changed to a novel object (● + ■), and mice were allowed to explore for 5 min. During both the familiarization and test phases, the time spent exploring each object was measured and recorded. 

All the neurobehavioral tests were recorded and analyzed with an image analyzing system (SMART v2.5.21 software and SMART video Tracking system (Panlab Harvard Apparatus, Barcelona, Spain).

### 4.4. Cell Culture and Drug Treatment

Microglia BV2 cells and PC12 cells were obtained from the American Type Culture Collection (ATCC, Manassas, VA, USA, #2469) and KCLRF, Korean Cell Line Research Foundation (KCLRF, Seoul, Korea, #21721), respectively. BV2 cells were cultured in PRMI 1640 containing 10% fetal bovine serum, penicillin (100 U/mL), and streptomycin (100 mg/L) at 37 °C in a humidified environment with 5% CO_2_. To activate NLRP3 inflammasome, BV2 cells were sub-cultured at a density of 1 × 10^6^ cells/mL in six-well plates and incubated with lipopolysaccharide (LPS, 1 mg/mL, Sigma-Aldrich, Burlington, MA, USA) for 3 h (for NLRP3 priming), followed by the addition of 5 μM concentrations of Adenosine triphosphate (ATP) (Sigma-Aldrich) for 30 min [13].

To determine optimum doses of Dex in vitro study, BV2 cells were treated with various concentrations of Dex (0, 5, 10, 20, 40, 60 μg/mL) for 24 h, and the cell viability was measured using colorimetric MTS assay. To determine the protective effect of Dex, cells were pretreated with 20 μg/mL of Dex for 24 h before exposing them to LPS and ATP. PC12 cells were exposed to the conditioned medium (CM) from BV2 cells treated with LPS and ATP after differentiation as follows: differentiation of PC12 cells was induced in DMEM containing 1% FBS, 0.25% BSA, and 50 ng/mL NGF for six days followed by replacing the medium with CM (1% FBS) with 0.25% BSA and 50 ng/mL NGF. PC12 cells were further cultured for four days for morphological analysis. PC12 cells were cultured as described previously with some modifications [52]. 

### 4.5. Western Blotting and Immunoprecipitation (IP)

Cells were washed using PBS and lysed using the protein lysis buffer (M-per) to obtain protein lysates. Equal amounts (30 μg) of proteins were loaded and separated using SDS-PAGE electrophoresis. The following primary antibodies were used: NLRP3 (Cell Signaling Technology, Danvers, MA, USA, 15101S, 1:1000), caspase-1 (Millipore, Burlington, MA, USA, AB1871, 1:1000), IL-1β (Cell Signaling Technology, 52718, 1:1000), β-Actin (Cell Signaling Technology, 4970S, 1:5000). β-actin was used as a loading control, and the density of bands was analyzed using the ImageJ software (NIH, Bethesda, MD, USA). 

The IPs were performed as previously described [53]. Briefly, the lysates were preincubated with 1 μg rabbit anti-NLRP3 antibody (Cell Signaling Technology, D4D8T) overnight at 4 °C. Subsequently, 20 μL of protein G Dynabeads (Invitrogen, Carlsbad, CA, USA, 10003D) were added, followed by incubation at 4 °C for 4 h and centrifugation for 5 min at 3000× *g*. The pellet obtained was washed using RIPA buffer and resuspended in 2× loading buffer. Samples were boiled to elute the protein complexes from the beads. NLRP3 antibody (Cell Signaling Technology, 15101S) was used for normalizing the loading amount. IgG was used as the negative control.

### 4.6. TUNEL Staining and ROS Detection

The apoptotic PC12 cells were detected using the terminal deoxynucleotidyl transferase-mediated dUTP nick-end labeling (TUNEL) assay (Roche, Basel, Switzerland) according to the manufacturer’s instructions. After the reaction with DAPI for 60 min at 37 °C, the cells were mounted with VECTASHIELD (Vectorlab, Mowry Ave Newark, CA, USA, H-1000). 

Reactive Oxygen Species (ROS) production was measured using dihydroethidium (Het; Invitrogen, D1168, 100 μM), which is oxidized by intracellular ROS. PC12 cells were exposed to the CM from BV2 cells for 16 h, and then cells were incubated with 100 μM Het for 30 min. The cells were washed with PBS and fixed with 4% paraformaldehyde. The cells were then stained with DAPI and mounted with VETASHIELD. Images were obtained using a fluorescence microscope (Olympus, Osaka, Japan).

### 4.7. Enzyme-Linked Immunosorbent Assay (ELISA)

For the ELISA analysis, animals were fully anaesthetized and perfused with saline after the neurobehavioral test. The hippocampal tissue was obtained and stored at −80 °C before use. The tissues were lysed using tissue protein extraction reagent (T-PER, Thermo Scientific, Waltham, MA, USA) containing protease and phosphatase inhibitor cocktail (Thermo Scientific,) and then homogenized and centrifuged at 13,000 rpm for 10 min to obtain the sample supernatants. Supernatant protein concentrations were measured with a BCA Protein Assay Kit (Thermo Scientific) according to the manufacturer’s specifications. NLRP3 inflammasome components and TNF-α, IL-18, and IL-1β were assayed using ELISA and commercially available kits, according to the manufacturer’s protocol (R&D Systems, Minneapolis, MN, USA). In some cases, the levels of IL-1β were measured from the supernatant of the cell free culture medium according to the manufacturer’s specifications. 

### 4.8. Cell Viability Assay

A colorimetric MTS assay was performed using 96 AQueous CellTiter according to the manufacturer’s protocol with some modifications. Briefly, CellTiter96 Aqueous (1/5 volume of the medium) was added to the cell cultures, plated in 24-well plates. The total absorption at various time points was measured using an ELISA microplate reader (VersaMax, Molecular Devices, San Jose, CA, USA) with the absorption wavelength set at 490 nm.

### 4.9. Statistical Analysis

Data are expressed as mean ± SME. Statistical comparisons between multiple groups were made using ANOVA followed by Tukey’s post hoc test (SPSS, version 5.01; SAS Institute Inc., Cary, NC, USA). Results between two independent groups were compared using *t*-test or Mann–Whitney U test. The level of significance was set at * *p* < 0.05

## 5. Conclusions

The neuroprotective effect of Dex on the POCD condition is associated with NLRP3 inactivation. This study also reports the neuroprotective effects of Dex against oxidative stress-related neuronal death induced by NLRP3 inflammasome activation in POCD condition.

## Data Availability

The data supporting the findings of this study are presented within the manuscript.

## Abbreviations

ASC	Apoptosis-associated speck-like protein
ATP	Adenosine triphosphate
CM	Conditioned medium
Dex	Dexmedetomidine
ELISA	Enzyme-linked immunosorbent assay
EPM	Elevated plus maze
Het	Dihydroethidium
IL-1β	Interleukin (IL)-1-beta
IL-6	Interleukin (IL)-6
IP	Immunoprecipitation
LPS	Lipopolysaccharide
NLR	NOD-like receptor
NLRP3	The NOD-like receptor pyrin domain-containing 3
NOD	Nucleotide-binding oligomerization domain
NORT	Norvel objective recognition test
OFT	Open field test
POCD	Postoperative cognitive dysfunction
ROS	Reactive oxygen species
SMA	Superior mesenteric artery
TNF-α	Tumor necrosis factor-alpha
TUNEL	Terminal deoxynucleotidyl transferase-mediated dUTP nick-end labeling
YOH	Yohimbine hydrochloride
